# Balint Syndrome in a Patient With Isolated Corpus Callosum Stroke: A Case Study With Narrative Review

**DOI:** 10.7759/cureus.85402

**Published:** 2025-06-05

**Authors:** Tom Changlai, Bertrand Liang

**Affiliations:** 1 Neurology, St. Joseph's Medical Center, Stockton, USA; 2 Neurology, University of Colorado School of Medicine, Aurora, USA

**Keywords:** attention, balint syndrome, corpus callosum stroke, neglect, neural networks (nns), visual working memory

## Abstract

We report a case of an isolated bilateral corpus callosum stroke (body and splenium) who exhibited Balint syndrome (optic ataxia, simultanagnosia, optic apraxia), along with a partial Gerstmann syndrome (left/right disorientation, acalculia, finger agnosia), with extinction to double simultaneous stimulation and astereognosis without direct involvements of the parietal lobes. We review the vascular anatomy of the corpus callosum, mechanisms and risk factors for stroke in this area, and the components of Balint syndrome and localization, with descriptions of recent data on neural networks involved in higher-level cortical function. Attention to the visual-spatial symptoms of Balint syndrome is important to consider when evaluating and treating patients with ischemic disease of the corpus callosum.

## Introduction

Balint syndrome is a rare neurological condition that has been described as resulting from damage to both the parietal lobes of the brain, often in the occipital-parietal junction area. The connections in this area of the brain are thought to play a role in processing visual information and spatial awareness. The initial description by Balint who evaluated patients who were soldiers with bilateral parietal lobe lesions [1] noted specific clinical manifestations, including optic ataxia (difficulty guiding movements toward visual targets, viz., reaching for an object based on visual (cf. proprioceptive) information), visual simultanagnosia (inability to perceive more than one visual object at a time, i.e. being able to focus on only one object in a scene rather than the whole without a deficit in visual fields), and optic apraxia (inability to shift gaze voluntarily to objects of interest despite intact extra-ocular movements). Like the original description, ischemic disease has been reported as a frequent etiology of this syndrome, with hemorrhage, traumatic brain injury, cancer, and neurodegenerative diseases, as well as N-methyl-d-aspartate receptor (NMDAR) encephalitis associated with the syndrome [2].

Higher-order cognitive symptoms can result from ischemic disease(s). Isolated corpus callosum (CC) stroke is uncommon, occurring in less than 1% of patients with ischemic disease [3]; most often, CC stroke is associated with other areas of ischemia in the ipsilateral or contralateral hemisphere [3,4]. It is likely that the low prevalence of isolated CC stroke is due to the dual blood supply of both the anterior and posterior circulation and the density of the white matter tracts of the CC [5]. Involvement of the CC typically affects higher-order alterations in brain function, including findings such as apraxia, agraphia, tactile anomia/astereognosis of the left hand, and alien hand syndrome [6].

We report a case of a patient with isolated splenium and body CC stroke with associated Balint syndrome, combined with other components of neglect, viz., a partial Gerstmann syndrome (acalculia, left-right disorientation, finger agnosia) and astereoagnosia/extinction. We review the vascular anatomy for the CC, as well as the localization of components of Balint syndrome; discuss the functional neural networks described; and postulate the involvement of the connections at the CC, mimicking the involvement of bilateral parietal lobes affecting a complex higher brain function syndrome. This case contributes to current knowledge and adds to the existing data around the neuroanatomical basis of Balint syndrome.

## Case presentation

A 30-year-old man with a history of end-stage renal failure on hemodialysis (MWF) and diabetes was initially brought to the emergency department due to bilateral lower extremity and abdominal wounds with pain after completing dialysis. The patient had a history of chronic hypotension and was being treated with oral vasopressors (midodrine hydrochloride). Upon initial evaluation, blood pressure was 89/46 mmHg, with a pulse rate of 98 beats per minute, a respiratory rate of 20 breaths per minute, and the patient being afebrile. General examination revealed abdominal and lower extremity wounds, with a neurologic exam being nonfocal. Lab examination was notable for a leukocytosis of 13.6 cells/mm^3^, hemoglobin of 8.0g/dL, sodium of 132 mEq, potassium of 5.2 mEq, creatinine of 7.0 mg/dL, total bilirubin of 3.4 mg/dL, and alkaline phosphatase of 505 IU/L. The patient underwent a CT abdomen, which revealed abdominal wall cellulitis without abscess; chest X-ray showed vascular congestion and cardiomegaly. Ultrasound of the lower extremities did not reveal any deep vein thrombosis.

The patient was begun on vancomycin for sepsis and norepinephrine bitartrate to support blood pressure. However, the patient had persistent hypotension, and antibiotic coverage was broadened to include additional cefepime and clindamycin. Two days later, the patient was found to have an altered mental status with left-sided “weakness.” Neurologic examination at that time revealed left/light disorientation, finger agnosia, and acalculia. The patient exhibited difficulty in moving gaze from one object to another, despite having intact oculomotor function, and could not describe objects (pen, coin - a US quarter) within the intact visual fields, unless one object was removed. The patient could not reach for an object on the left, particularly with the left hand, when the object was placed in either the left or right visual field, although the patient was not hemiparetic. The patient would move the left upper extremity fully when the right was also stimulated, but there was a paucity of movement otherwise. Extinction was noted to double simultaneous stimulation to the right, with loss of stereognosis in the left hand. No alterations in cranial nerves, motor function, or sensory function were noted; there were two beats of clonus at the ankle on the right.

**Figure 1:**
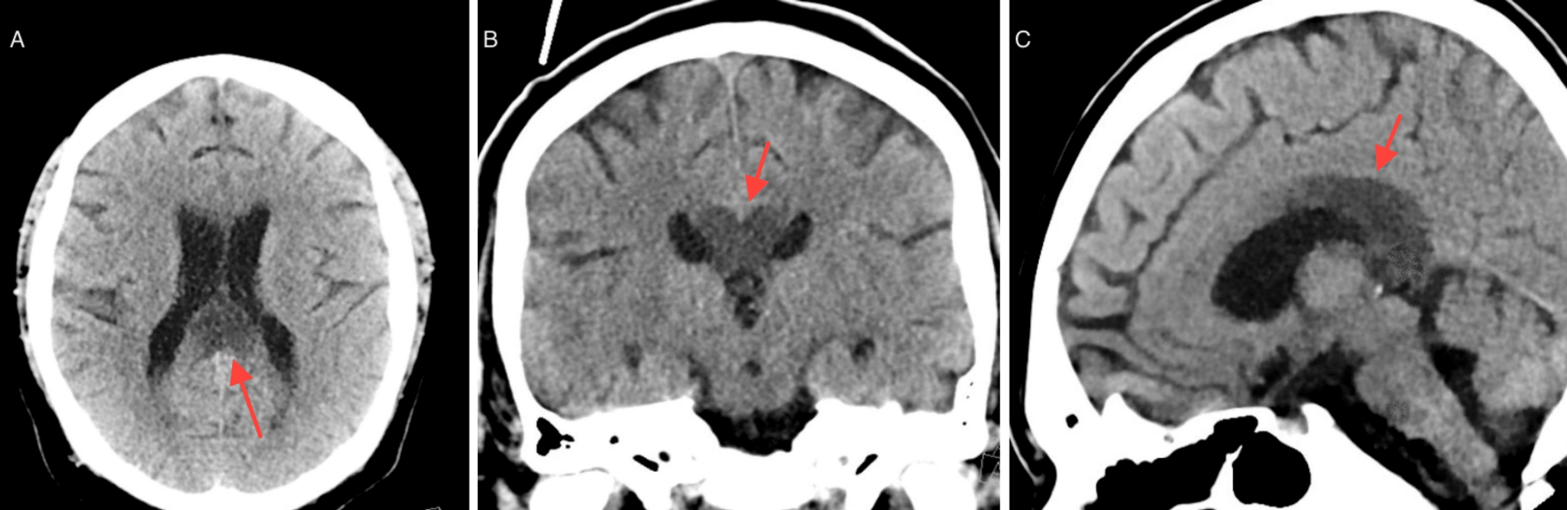
CT scan. A. Axial view. B. Coronal view. C. Sagittal view. Red arrows show the areas of ischemia.

**Figure 2:**
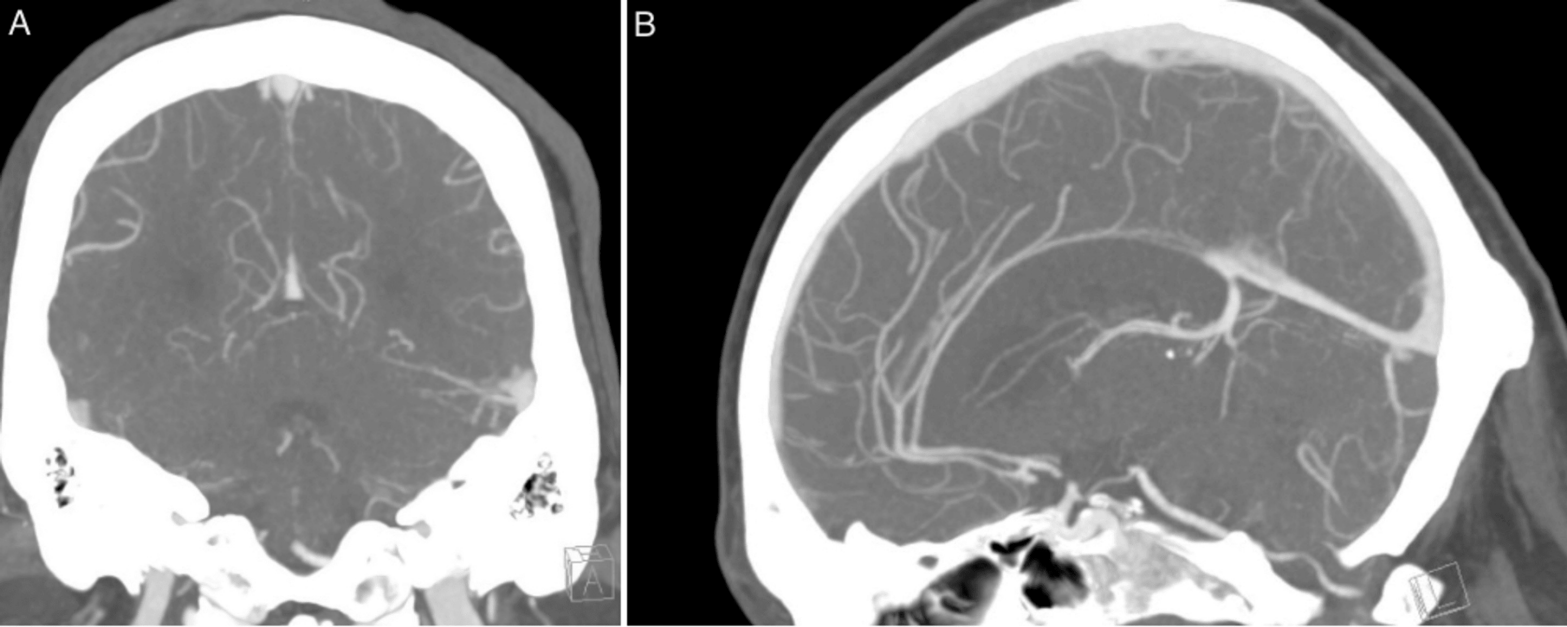
CT angiogram. A. Coronal view. B. Sagittal view. No abnormalities were detected on CT angiogram.

A CT scan (Figure 1) showed evidence of ischemia at the body and splenium of the corpus callosum bilaterally, with mild-to-moderate chronic microvascular ischemic changes. A CT angiogram (Figure 2) was performed, which revealed no large vessel occlusion nor any significant intracranial/neck vascular abnormality.

The patient was begun on oral daily aspirin (81 mg). However, the patient began having worsening control of blood pressure, with evidence of sepsis and a cutaneous mucormycosis, and was begun on amphotericin B. Repeat abdominal CT revealed abscesses at the site of the initial infection, and the patient underwent incision and drainage of these lesions. One day later, the patient became tachycardic and hypotensive once again and was found to have worsening leukocytosis; the patient entered into ventricular fibrillation, and despite active resuscitation efforts, the patient expired.

## Discussion

Corpus callosum stroke

Vascular Supply 

Isolated CC stroke is an uncommon entity and is typically associated with involvement of other areas of the cortex. In case series, most often the area involved is the splenium, with the body and the genu following in frequency [7]. All series note the infrequency of isolated involvement of the CC as related to the dense vascular supply, which includes the anterior communicating artery (ACA), the pericallosal artery, and the posterior pericallosal artery [8]. Indeed, the rostrum and genu are supplied by the subcallosal and medial callosal artery from the ACA, with the pericallosal artery from the ACA giving rise to four branches providing blood supply to the CC body. The posterior pericallosal artery from the posterior cerebral artery (PCA) provides blood supply to the splenium, and there are numerous anastomoses between the ACA and PCA, particularly near the inferior aspect of the splenium [8]. Moreover, the density of the white matter tracts may result in “physiologic reserve,” allowing for some ischemia to remain relatively asymptomatic, although this is speculative [5,7]. In addition, it has been hypothesized that the perpendicular orientation of the callosal branches may prevent embolization of the vascular supply to the CC [7]. In various series, a combination of infarction of the splenium and body was only rarely encountered [6,7], particularly in isolation, which nonetheless occurred in our patient.

Clinical Features

Because of the rarity of CC stroke, case series provide data on clinical experience. In general, clinical features of isolated CC stroke have been associated with mild hemiparesis, sensory changes including decreased sensation contralaterally, and alien hand syndrome and neglect [3]. Other symptoms include vertigo, dysarthria, and ataxia, as well as aphasia (often incomplete) and dyskinesia, with one case reported with “grope for action” limb findings/limb apraxia (a presumed manifestation of alien hand syndrome) [3,6,7]. Importantly, much of the experience combines the involvement of the CC with other areas of the brain, and as a result, clinical syndromes observed are more likely to be mixed. Notably, in our patient with isolated bilateral CC stroke of the body/splenium, it was higher cortical functions that were involved rather than sensory or motor findings.

Risk Factors

Risk factors of CC stroke are similar to those of ischemic stroke in general. Hypertension, hyperlipidemia, diabetes, cerebral artery stenosis, smoking, and coronary artery disease are uniformly reported in case studies. In one of the largest case series, particularly of isolated CC stroke, Li et al. [3] compared different areas of isolated infarction of the CC. In their experience, no differences were noted with respect to age and sex in the genu/body vs. splenium locations. However, there was an increased frequency of atheroma in source arteries (internal carotid, vertebrobasilar artery, respectively) in genu/body vs. splenium infarctions. Splenium infarcts were more often bilateral compared to those in the genu/body in that study. Notably, in a smaller case series, this was not seen [9]. In concert with these findings, our patient manifested bilateral isolated infarcts in the body and splenium and had a history of hypertension and diabetes.

Balint syndrome

Localization

The initial description of Balint syndrome was in a patient found to have bilateral parietal lobe insults [1]. However, as opposed to Gerstmann syndrome, where the localization to the most often left angular gyrus has been consistently established, the degree of specificity to the area(s) of the brain responsible for Balint syndrome is inconsistent and diverse [10]. Indeed, the syndrome has been associated with lesions of the bifrontal cortex, pulvinar lesions, lesions of Brodmann areas 6 and 8, as well as involvement of areas 5, 7, 19, 37, and 39 and the mesial right temporo-occipital areas [10]. Moreover, the different components of Balint syndrome have been localized to different areas/connections of the brain, both cortical and subcortical, as manifestations of the higher brain functions, which are assumed to be involved [2].

Simultanagnosia

It is thought that the visual mechanisms for object localization and recognition are actually processed outside of the visual system, including areas such as the cerebellum and networks in the frontal lobe [11,12]. There is ongoing debate on whether the visual agnosia of simultanagnosia is due to hemineglect vs. a differing independent syndrome of inattention [10,13]. An overlap exists in symptoms of hemineglect and Balint syndrome (e.g., attention toward only one aspect within the visual field with inattention to another), and it is noted that the former is typically associated with only right-sided parietal lesions. Balint syndrome canonically requires both sides [14]. Regardless, the symptom is associated with a presumed decline in attention, whether from a damaged parietal system or one connected to other, more diverse central nervous system networks [2,14].

Optic Ataxia

Optic ataxia has, in general, been localized to the superior parietal lobule and intra-parietal sulcus. Imaging data have shown that these areas are involved bilaterally and are the target of certain attention networks [14], which direct cues by which motor and sensory outputs are sequenced. This includes both the perception of an object, targeting and initiating and stopping movement. There is some controversy on whether the optic ataxia is a purely visual disorder or whether this is yet another attention-related syndrome [2,14]. In any case, bilateral brain involvement in Balint syndrome seems to be required for the finding in context with optic apraxia and simultanagnosia, more than just that of hemispatial neglect, which is often unilateral alone [2].

Optic Apraxia

Optic apraxia is closely related to simultanagnosia and is also considered an issue with attentional deficit. However, positron emission tomography (PET) studies suggest additional aspects that extend the higher brain functions further, which had been previously hypothesized, viz., to working memory [12]. Data from these studies reveal the importance of connections to the dorsolateral prefrontal areas, specific for working memory, particularly visual working memory for spatial localizations [11]. Further, the localization involves the symptom of lack of disengagement of attention from fixated objects [13], termed a “visual grasp reflex” [14]. As a result, overall, the findings of the connections of the parietal lobe both ipsilaterally and contralaterally seem to relate to visual working memory [15].

Brain Network Correlation

Hence, given the diverse nature of Balint syndrome, the involvement of both attention centers and visual working memory has been hypothesized as being relevant and required. Because these higher brain functions are integrated in both cortical tissue and central nervous system networks, and are bilateral in origin [16,17], the recent functional MRI (fMRI) data [18] are intriguing and of significant interest. These data reveal that the various attention domains recruit multiple and partly overlapping intrinsic networks and converge in the dorsal fronto-parietal and midcingulo-insular network bilaterally. This correlates to the need for connections between ipsilateral and contralateral areas of the cortex, thus requiring hemispheric crossing white matter tracts such as the CC.

Further, with regard to visual working memory, a similar bilaterality and localization is noted. In another fMRI study, Li et al. [19] noted that visual working memory was associated with increased connectivity within a frontal-opercular network, as well as between the dorsal attention network (intraparietal sulcus and frontal eye field localization) and an angular-gyrus-cerebellar network. These areas require, as does the attention intrinsic network, diverse ipsilateral and contralateral connections, particularly between the parietal lobe and the cingulate gyri. Indeed, it was found that coupling between the frontoparietal control network and the cingulo-opercular network contributes to visual memory precision.

As noted for attentional networks, the visual working memory neural correlates are underpinned by different yet partially overlapping intrinsic functional networks requiring significant connections. This includes bilateral cortical connections with fiber coherence of the bilateral superior longitudinal fasciculus and inferior fronto-occipital fasciculus. This once again suggests the relevance of bilateral connections, such as those of the CC [20].

Implications

In our patient with isolated CC stroke, the visual inattention and working memory aspects of Balint syndrome were present clinically, in addition to attention disorders of Gerstmann syndrome and extinction/astereognosis. Both these latter syndromes also typically localize to the parietal lobes, similar to that of the classically described Balint syndrome, although to the left and right, respectively.

What our clinical findings suggest is that the neural networks involved in these higher-order cognitive functions traverse through the CC, thus affecting the usual findings of parietal lobe involvement, both unilaterally and bilaterally, despite not having identified pathology within the lobes themselves. This is interesting as it adds to the burgeoning information on attention and memory networks within the human brain and the structural involvement in diverse functional networks of higher-order cognitive functions.

In addition, the clinical aspects of CC stroke, i.e., the visual attention and working memory issues, cannot be underestimated and need to be carefully considered when evaluating patients for treatment. The visual agnosia, optic ataxia, and ocular apraxia can be debilitating, particularly if not recognized and understood, especially whenever occurring with other motor or sensory symptoms. Indeed, patients (and providers) may not realize the presence of Balint syndrome symptoms; indeed, patients have been observed to be paralyzed due to the limitations of being unable to appreciate an entire visual scenario and/or inability to scan their visual fields even in familiar environments [14].

Clinicians caring for these patients need to be aware of these limitations due to CC stroke and address them in any rehabilitation effort. Indeed, these subtle cognitive signs, particularly in critically ill patients, need to be suspected when evaluating patients during hospitalization in order to address potential etiologies, treatments, and follow up in the outpatient setting.

## Conclusions

We report a patient with isolated CC stroke, involving the body and splenium bilaterally, who subsequently was found to have Balint syndrome along with a partial Gerstmann syndrome, extinction to bilateral simultaneous stimulation to the right, and astereognosis on the left, suggesting involvement of both parietal lobes despite no specific identified pathology. This case report of a Balint syndrome in an isolated bilateral CC stroke suggests that the CC is either/both engaged with or part of the neural networks involved in visual attention and working memory and has implications for the treatment of such patients in the rehabilitation setting. Further studies on human brain networks will be interesting to integrate these findings into current models of higher cortical functioning.
